# Anti-Tumor Effects of Carrimycin and Monomeric Isovalerylspiramycin I on Hepatocellular Carcinoma *in Vitro* and *in Vivo*

**DOI:** 10.3389/fphar.2021.774231

**Published:** 2021-11-26

**Authors:** Yong Jin, Hong Xiang Zuo, Ming Yue Li, Zhi Hong Zhang, Yue Xing, Jing Ying Wang, Juan Ma, Gao Li, Hongxin Piao, Puqing Gu, Xuejun Jin

**Affiliations:** ^1^ Key Laboratory of Natural Medicines of the Changbai Mountain, Ministry of Education, Molecular Medicine Research Center, College of Pharmacy, Yanbian University, Yanji, China; ^2^ Liver Diseases Branch, Yanbian University Affiliated Hospital, Yanji, China; ^3^ Shanghai Tonglian Pharmaceutical Co., Ltd, Shanghai, China

**Keywords:** carrimycin, monomeric isovalerylspiramycin I, hepatocellular carcinoma, anti-tumor, *in vitro*, *in vivo*

## Abstract

Hepatocellular carcinoma results in a high risk of second primary malignancies and has prominent morbidity and mortality. There is a lack of effective treatment and prognosis is poor. Therefore, effective drugs need to be discovered. Carrimycin is a 16-member macrolide antibiotic with anticancer activity, and monomeric isovalerylspiramycin I is a main component. The aim of this study was to determine the anti-tumor effects of carrimycin and monomeric isovalerylspiramycin I on hepatocellular carcinoma through *in vivo* and *in vitro* experiments. *In vitro*, changes in cellular proliferation, migration, invasion, and apoptosis were analyzed by MTT, colony formation, EdU labeling, wound-healing, matrigel transwell invasion, and flow cytometric assays using SK-Hep1, Hep3B, SNU-354, SNU-387 hepatocellular carcinoma cell lines. Western blotting and RT-PCR were used to detect the effects of carrimycin and monomeric isovalerylspiramycin I on the expression levels of vascular endothelial growth factor (VEGF) and programmed death ligand 1 (PD-L1). Nude mice were subcutaneously transplanted with SK-Hep1 cells or C57BL/6J mice were orthotopically transplanted with hepatocarcinoma H22 cells. Tumor volume, pathological changes in tumor tissues, and the concentration of VEGF in mouse serum were measured after treatments. Carrimycin and monomeric isovalerylspiramycin I dose-dependently inhibited hepatocellular carcinoma cell viability, colony formation, and DNA replication. These agents markedly suppressed migration and invasion and promoted apoptosis of the cell lines. Western blotting and RT-PCR demonstrated that carrimycin and monomeric isovalerylspiramycin I reduced VEGF and PD-L1 protein and mRNA levels in a dose-dependent manner. *In vivo* studies further confirmed that carrimycin and monomeric isovalerylspiramycin I could significantly inhibit tumor growth, tumor histopathological alterations, and the concentration of VEGF in both mouse tumor models. These results show that carrimycin and monomeric isovalerylspiramycin I promoted apoptosis and inhibited proliferation, migration, and invasion of hepatocellular carcinoma cells. Therefore, our discovery suggests anti-tumor capacity for carrimycin and monomeric isovalerylspiramycin I and provides data on potential new drugs for inhibiting hepatocellular carcinoma.

## Introduction

Hepatocellular carcinoma (HCC) is one of the leading causes of cancer-related death worldwide (El-Serag and Rudolph, 2007). Since the 1990s, the incidences of cirrhosis and hepatocellular carcinoma have increased dramatically in the Western world, and mortality from hepatocellular carcinoma has increased more rapidly than any other cancer (Ryerson et al., 2016). Cirrhosis and hepatitis B and C infections increase the risk of developing HCC. Although hepatitis B suppression or radical hepatitis C treatment can reduce the incidence of HCC, the therapeutic options for advanced HCC are limited (El-Serag, 2012). Chronic liver disease is the main risk factors for HCC. Treatment for HCC is usually limited because most patients with HCC are diagnosed at an advanced stage. Surgical resection, targeted therapy, immunotherapy, and systemic chemotherapy are among the current treatments for HCC. Due to molecular heterogeneity and chromosomal alterations, the molecular mechanism of HCC remain unclear, and therapeutic options still needed to be developed (Hoshida et al., 2010). HCC is known to be a highly vascularized solid tumor in which angiogenesis plays an important role in tumor progression, high recurrence rates, and poor survival. Vascular endothelial growth factor (VEGF) is a major growth factor that promotes HCC progression by regulating endothelial cell proliferation, migration, differentiation, and vessel formation (Pang and Poon, 2006). Additionally, HCC is characterized by its association with chronic inflammation and an immunosuppressive tumor microenvironment, which makes immunotherapy an approach for new drug development (Chang et al., 2017).

Tumor cells often overexpress immune checkpoint proteins, which allows evasion of the host immune system by inhibiting T-cell responses (Chang et al., 2017). One of these immune checkpoint proteins is programmed death ligand 1 (PD-L1), which binds to programmed death-1 (PD-1) expressed on the surface of T-cells, B-cells, dendritic cells, and natural killer T-cells and suppresses anticancer immunity (Goodman et al., 2017). The programmed cell death-1 (PD-1)/PD-1 ligand (PD-L1) pathway is an important immune checkpoint that contributes to the maintenance of self-tolerance and control of excessive immune responses (Koh et al., 2016). Recently, it has been shown that cancer cells also utilize the PD-1/PD-L1 pathway to evade host immune surveillance (Koh et al., 2016). Engagement of PD-1 by PD-L1 suppresses T-cell proliferation and function by inducing T-cell apoptosis, anergy, exhaustion, and the production of immune-suppressive cytokines (Kim et al., 2015). However, interactions between PD-1 and PD-L1 are reversible, and blockade of PD-1/PD-L1 interactions restores effector T-cell function and antitumor immune responses *in vivo* (Iwai et al., 2002; Koh et al., 2016). These findings suggest that blockade of the PD-1/PD-L1 pathway might affect immune-mediated tumor surveillance *in vivo*, enhancing tumor tumor cell death (Koh et al., 2016). It has been reported that PD-L1 expression is increased in HCC compared with normal liver tissue (Xiao et al., 2019). Furthermore, it has been shown that PD-L1 expression by neoplastic and inflammatory cells in the tumor microenvironment of HCC is significantly correlated with markers of tumor aggressiveness, and increased PD-L1 expression in HCC is associated with poor prognosis and disease recurrence (Aoki et al., 2020). Therefore, it is a new idea to treat tumors by inhibiting PD-L1 expression.

Traditional chemotherapy drugs include alkylating agent, an-timetabolic drugs, hormone drugs, platinum drugs, botanical drugs, an-tibiotic drugs, molecular-targeted drugs, and so on, which are usually combined to exert anti-tumor effect. Because of malnutrition, immune barrier breakdown induced by radiotherapy and chemotherapy, and is-chemic necrosis in tumor center, patients with malignant tumor are more likely to be involved in perioperative infection. Thus, antibiotic drugs may play a dual role in anti-infection and anti-tumor functions, indicating its importance in cancer treatment. However, there are also apparent systemic toxicities of current anti-tumor antibiotics, for exam-ple, cardiotoxicity and bone marrow suppression of anthracycline drugs, pulmonary fibrosis induced by bleomycin and cytotoxicity of human erythrocyte caused by macrolides (Chen and Stubbe, 2005; Cagel et al., 2017; Eloy et al., 2017). Thus, there is an urgent need of finding new drugs with both good anti-tumor activity and low biotox-icity.

Carrimycin is a genetically engineered drug that was developed by synthetic biological technology and belongs to the macrolide class of antibiotics. The main components of carrimycin are isovalerylspiramycin I, II, and III with minor components consisting of butyrylspiramycin, propionylspiramycin, and acetylspiramycin II and III (Guangdong et al., 2001). Carrimycin is a broad-spectrum antibacterial agent and is effective against Gram-positive cocci, β-lactamase producing bacteria, *Mycoplasma* and *Chlamydia* spp., as well as some Gram-negative bacilli and fungi (Liang et al., 2021). Completed phase III clinical trials showed that carrimycin was safe, required a low oral dose, had a low adverse reaction rate, and there was no secondary drug resistance (Guangdong et al., 2001). Additionally, it has been reported that carrimycin can be used in the treatment of COVID-19 with good therapeutic effect (Lima et al., 2020). Furthermore, our preliminary experiments showed that carrimycin functioned as a regulator of mammalian cell proliferation. As we all know, cancer is closely related to inflammation, and inflammation can lead to the occurrence and development of cancer. So we speculate that carrimycin may also inhibit tumor growth as an anti-inflammatory drug. Previous studies have shown that immunosuppressor rapamycin (macrolides antibiotics) inhibits tumor progression through targeting mammalian target of rapamycin (mTOR) (Foster and Fingar, 2010). Epothilone binds to microtubulin to suppress mitosis, and its analogs were reported to down-regulate the phosphorylation of AKT, ERK and p38 (Giannakakou et al., 2000; Edelman and Shvartsbeyn, 2012; Li et al., 2016). Since there’s little study reporting the possible mechanism of carrimycin on tumor inhibition, based on the theories mentioned above, we hypothesize that carrimycin may exert anti-tumor function. We prove for the first time carrimycin and monomeric isovalerylspiramycin I inhibited VEGF and PD-L1 protein expression and tumor growth of hepatocellular carcinoma.

Lenvatinib (LEN) is an inhibitor of tyrosine kinase receptors, including VEGF receptor, fibroblast growth factor receptor, platelet-derived growth factor receptor, and rearranged during transfection (also known as RET) proto-oncogene (Matsui et al., 2008b; Okamoto et al., 2013). This drug has anti-angiogenic activity and a direct anti-proliferative effect on tumor cells by preventing relevant signaling pathways (Matsui et al., 2008a; Matsui et al., 2008b; Glen et al., 2011). Lenvatinib can be administered orally, and promising anticancer effects have been observed in phase III clinical trials for hepatocellular carcinoma. Lenvatinib has brought clinical benefits for patients; however, adverse events are inevitable and include hypertension, fatigue, proteinuria, nausea, decreased weight, and abdominal pain, which may decrease the quality of life of patients and influence their acceptance of treatment.

There is a strong need to find safer and effective alternative drugs for the treatment of HCC. Our study examined the effects of carrimycin and monomeric isovalerylspiramycin I on proliferation, apoptosis, invasion, migration, and expression of VEGF and PD-L1 in hepatocellular carcinoma cells. Lenvatinib was used as a positive control drug in our study. In addition, the effects of carrimycin and monomeric isovalerylspiramycin I were investigated *in vivo* using xenograft and orthotopic mouse tumor models.

## Materials and Methods

### Cell Culture and Reagents

SK-Hep1, Hep3B, and SNU-354 cells were grown in DMEM with penicillin (100 U/mL)-streptomycin (100 U/mL) and 10% heat-inactivated fetal bovine serum. SNU-387 cells were cultured in RPMI-1640 medium supplemented as above. All cells were cultured in a cell incubator at 37°C, 5% CO_2_, and appropriate humidity. Hepatocellular carcinoma cells were purchased from the Shanghai Cell Bank of the Chinese Academy of Sciences. Dimethyl sulfoxide (DMSO) was purchased from Sigma Chemical Co. Carrimycin (batch number:20180625), monomeric isovalerylspiramycin I (batch number:20180517) and, lenvatinib (purity:99.67%) were provided by Shangke Biopharmaceutical (Shanghai) Co., Ltd.

*In vitro* test solution: carrimycin (3, 10, 30 μg/mlL), monomeric isovalerylspiramycin I (1, 3, 10 μg/ml), and lenvatinib (3, 10 μg/ml).

### Measurement of Cell Proliferation by MTT Assay

SK-Hep1, Hep3B, SNU-354, and SNU-387 cells were seeded at a density of 1×10^5^ cells/well in 96-well plates and incubated overnight. The cells were pretreated with carrimycin (0.001, 0.003, 0.01, 0.03, 0.1, 0.3, 1, 3, 10, 30 μg/ml), monomeric isovalerylspiramycin I (0.001, 0.003, 0.01, 0.03, 0.1, 0.3, 1, 3, 10, 30 μg/ml), and lenvatinib (0.001, 0.003, 0.01, 0.03, 0.1, 0.3, 1, 3, 10, 30 μg/ml) for 24, 48, 72 h. At the end of the experiment, the medium was removed and cells were cultured with MTT solution (5 mg/ml) [3-(4,5-dimethylthiazol-2-yl)-2,5-diphenyltetrazolium bromide] (Sigma, St. Louis, MO, United States) for 4 h. The viable cells converted MTT to formazan, which generated a blue-purple color after dissolving in 100 μl of DMSO. The absorbance at 570 nm was measured by Multiskan GO.

### Colony Formation

SK-Hep1, Hep3B, SNU-354, and SNU-387 cells were seeded in 6-well plates (5×10^3^ cells/plate) for colony formation assay. Following 24 h incubation, the cells were treated with various concentrations of carrimycin, monomeric isovalerylspiramycin I, and lenvatinib. Cells treated with DMSO were used as negative controls. After 10 days, the cells were washed with PBS. The colonies were stained with 1% crystal violet for 30 s after fixation with 10% formaldehyde for 5 min. The dishes were rinsed three times with PBS and air-dried.

### EdU Labeling and Immunofluorescence

EdU is a thymidine analog that is incorporated into replicating chromosomal DNA during the S phase of the cell cycle. We used the EdU incorporation assay, which is a highly sensitive and specific method, to investigate the inhibitory effects of carrimycin, monomeric isovalerylspiramycin I, and lenvatinib. Cells were cultured in 96-well plates for 24 h, and then cells were treated with carrimycin, monomeric isovalerylspiramycin I, and lenvatinib for 16 h. Next, the cells were labeled with 5-ethynyl-2′-deoxyuridine (EdU, RIBOBIO; Guangzhou, China) for 6 h, the cells were washed with PBS for 2 min. After being fixed with 4% paraformaldehyde for 30 min, the cells were decolorized with 2 mg/ml glycine for 3 min, treated with 0.5% Triton X-100 for 10 min, and rinsed with PBS three times. Thereafter, the cells were exposed to 100 μl of 1×Apollo^®^ reaction cocktail for 30 min and incubated with 5 μg/ml of Hoechst 33,342 to stain the cell nuclei for 30 min (Mi et al., 2017). The stained cells were observed with Olympus IX83 inverted fluorescence microscope (Olympus Corporation, Tokyo, Japan).

### Wound-Healing Assay

SK-Hep1, Hep3B, SNU-354, and SNU-387 cells were seeded in 24-well plates (1×10^5^ cells/plate) for wound-healing assay. The cells were cultured to form a monolayer and wounds were made by scratching with a pipette tip and exposed with different treatments for 0, 24, 48, and 72 h. The cell migrations were photographed at identical locations and analyzed by comparing the final gap width to the control group with Olympus IX83 inverted fluorescence microscope (Olympus Corporation, Tokyo, Japan).

### Matrigel Transwell Invasion Assay

SK-Hep1, Hep3B, SNU-354, and SNU-387 cells were seeded in a 6 cm Petri dish for matrigel transwell invasion assay. After the cells were attached, the cells were treated with various concentrations of carrimycin, monomeric isovalerylspiramycin I and lenvatinib, and continued to cultivate for 4 days. Matrigel (BD Biocoat, Bedford, MA, United States) was diluted with a serum-free medium to a final concentration of 1 mg/ml, 100 μl of the diluted matrigel was added vertically to the bottom center of the upper chamber of the transwell, and incubated at 37°C for 1 h to make it dry into a gel. After washing the cells with PBS, the cells were suspended in serum-free medium and counted, then adjusted the concentration to 1 × 10^6^/ml, added 100 μl of the cell suspension to the upper chamber and 600 μl of medium containing 10% FBS to the lower chamber (bottom of the 24-well plate). After 24 h of invasion at 37°C, cells passing the filters into bottom wells were fixed with paraformaldehyde and stained with 0.1% crystal violet. Invaded cells were photographed by a microscope.

### Cell Apoptosis Analysis

SK-Hep1, Hep3B, SNU-354, and SNU-387 cells were seeded in 6-well plates (5×10^3^ cells/plate) for cell apoptosis analysis. After the cells were attached, the cells were treated with various concentrations of carrimycin, monomeric isovalerylspiramycin I, and lenvatinib. After 48 h of treatment, the cells were collected in a 1.5 ml tube, washed with PBS for 3 times, centrifuged to discard the supernatant, washed with binding buffer, centrifuged to discard the supernatant, the cells were resuspended in 50 μl binding buffer, and stained with Annexin V-FITC for 30 min and propidium iodide for 10 min at 37°C in the dark. The samples were analyzed by flow cytometry. The CellQuest software was used to analyze the data (Becton-Dickinson).

### Western Blotting

SK-Hep1, Hep3B, SNU-354, and SNU-387 cells were seeded in a 6 cm Petri dish for western blotting. After the cells were attached, the cells were treated with various concentrations of carrimycin, monomeric isovalerylspiramycin I, and lenvatinib for 12 h. The cells were washed with pre-cooled PBS and lysed on ice for 30 min. The lysate was collected, centrifuged at 14,000 rpm and 4°C for 30 min, and then the supernatant was collected to obtain cell extracts. The total protein concentrations were measured by the Bradford method. The cell extracts were separated by SDS-polyacrylamide gels (8–10%) and then the protein was transferred to a polyvinylidene difluoride membrane (Millipore, Bedford, MA, United States). The membrane was blocked with 5% skim milk, and incubated with the PD-L1 (Novus Biologicals, NBP1-76769, 1:2,000) and VEGF (Santa Cruz, Cat# sc-152, 1:500) primary antibody (The antibodies were diluted using 1% BSA in PBS). After binding of rabbit secondary antibody (Santa Cruz, Cat# sc-2004, 1:2,000) coupled to horseradish peroxidase, proteins were visualized by enhanced chemiluminescence according to the manufacturer’s instructions (Amersham Pharmacia Biotec, Buckinghamshire, United Kingdom).

### Semi-Quantitative RT-PCR Analysis

Total RNA was extracted using a Trizol reagent (Invitrogen). cDNA synthesis was executed with the Transcriptor First Strand cDNA Synthesis Kit (Roche, Basel, Schweiz) and then amplified by TaKaRa TaqTM RT-PCR kit (TaKaRa). The cycling conditions were 94°C for 4 min, followed by 40 cycles of 94°C for 30 s, 58°C for 30 s, 72°C for 30 s, and final conditions was 72°C for 5 min. The PCR products were electrophoresed on 3% agarose gels and stained with ethidium bromide. The stained bands were visualized under UV light.

### *In Vivo* Xenograft Assay

BALB/c athymic nude mice (male, 4–5 weeks old, 22 ± 2 g and specific-pathogen-free) were purchased from BEIJING HFK BIOSCIENCE CO., LTD (Beijing, China). The animals were treated humanely, and all efforts were made to minimize the animal suffering and numbers of experimental animals. The animals were given standard pellet food and water ad libitum and subjected to a 12 h light/12 h dark cycle. The room temperature was 19–23°C, and humidity was 35–55%. Lenti-EGFP virus particles were used to transfect SK-Hep1 cells (5×10^7^ cells/mL). The transfected cells (0.1 ml) were subcutaneously injected into the mice on the left side of the back. After 7 days of injection, the tumors formed on the back of the mice. The mice were randomly assigned to nine groups (n = 9/group). The volume of each administration is 200 μl, the carrimycin concentration is 2, 4, 8 μg/ml. The monomeric isovalerylspiramycin I concentration is 1, 2, 4 μg/ml. The lenvatinib concentration is 1 μg/ml. Carrimycin, monomeric isovalerylspiramycin I, and lenvatinib were orally given to the mice once a day for 28 days. The blank control group and the model group were given the same volume of solvent dissolved in carrimycin. The sizes of the tumors were measured every 2 days. The volume of the tumor was calculated by using the equation: volume of the tumor (mm^3^)=(width)^2^ × length/2. After 28 days, mice died peacefully after intraperitoneal injection of barbiturates, tumors were excised and fixed to conduct further experiments. All the protocols have obtained approval from the Committee of Animal Experiments Ethics in Yanbian University [(SPF, SCXK [JI] 2017-0003), Jilin, China].

### Orthotopic Liver Transplantation Assay

All the procedures and operations in animal experiments had adhered to IACUC guidelines. C57BL/6J mice (male, 4–5 weeks old, 20 ± 2 g and specific-pathogen-free) were purchased from BEIJING HFK BIOSCIENCE CO., LTD (Beijing, China) and kept in the experimental animal center of Yanbian University authorized by the Chinese Association for Laboratory Animal Sciences (approval ID: SCXK (Jinlin) 2017-0003) during the experiment. The animals were treated humanely, and all efforts were made to minimize the animal suffering and numbers of experimental animals. The animals were given standard pellet food and water ad libitum and subjected to a 12 h light/12 h dark cycle. The room temperature was 19–23°C, and humidity was 35–55%.The mice were randomly assigned to nine groups (n = 9/group). H22 tumor cells were cultured, collected, and centrifuged to adjust the cell concentration to 1 × 10^7^/ml. After disinfecting the abdomen of C57BL/6J mice with alcohol cotton balls, the mice were injected intraperitoneally with 0.1 ml H22 cell suspensions, and the changes of the mice abdomen were observed every day. After 7–10 days, a large amount of ascites was formed in the abdomen of the mice. We used disposable syringes to extract ascites cells from mice, the cell concentration was adjusted to 1 × 10^7^/ml with sterile PBS buffer, and cells suspension were injected intraperitoneally to second-generation mice. After repeating the operation three times, the ascites cells were collected and washed twice with saline, adjusted the cell concentration to 2 × 10^7^/ml, three generations of ascites cells were collected for subsequent use.

### Animal Model Establishment

C57BL/6J healthy mice fasted for 10 h and water-free for 4 h before surgery. The mice were anesthetized intraperitoneally with 4% chloral hydrate at a dose of 0.01 ml/g. The mice were fixed, and their skins were routinely disinfected and flattened. An incision of about 1 cm was made under the xiphoid process in the middle of the upper abdomen, and the skin and peritoneum were cut layer by layer, arranged the gauze soaked with saline under the incision, gently squeezed both sides of the costal arch. Exposed the left lobe of the mouse liver to wet gauze, and used 50 μl micro syringes to insert the needle along the long axis of the liver lobe at an angle of 20°C parallel to the liver surface. After inserting the needle, moved about 0.5 cm in parallel, 50 μl containing 3 × 10^6^ H22 cells were slowly injected into the mouse liver. With drawn the needle, gently pressed the needle eye with a cotton swab, and gently wiped the area around the needle eye with an alcohol cotton ball to remove a small number of leaked tumor cells and prevented tumor cells from escaping. Gently returned the liver to the abdominal cavity. After checking for active bleeding, sutured the abdominal wall layer by layer with No. One silk thread, disinfected the skin again, and closed the operation. After 4 h of fasting and water prohibition, 0.3% sucrose water was given, and 12 h later, they were given with normal food and water.

After the model was established, the mice were randomly divided into nine groups: blank control group, model group, carrimycin 20, 40, 80 mg/kg group, monomeric isovalerylspiramycin I 10, 20, 40 mg/kg group and the positive control drug lenvatinib 10 mg/kg group (n = 12/group). Carrimycin, monomeric isovalerylspiramycin I, and lenvatinib were orally given to the mice once a day for 14 consecutive days. The volume of administration was 5 ml/kg. The blank control group and the model group were given the same volume of solvent dissolved in carrimycin.

After 13 days, mice died peacefully after intraperitoneal injection of barbiturates, the livers were dissected and taken out, the tumor tissues were separated, weighed, and photographed. Tumors were fixed to conduct further experiments. All the protocols have obtained approval from the Committee of Animal Experiments Ethics in Yanbian University.

### Histological Analysis

Liver tissues and colon tissues were fixed in 4% formaldehyde, embedded in paraffin blocks, and cut into 5 μm tissue specimens. The sections were stained with hematoxylin and eosin (H&E) according to a standard protocol, and examined under a light microscope (ECLIPSE Ni-U, Nikon, Japan).

### Enzyme Linked Immunosorbent Assay Analysis

The concentrations of mouse VEGF were measured using ELISA kits (Shanghai Enzyme-Linked Biotechnology Co., Ltd. LOT: 07/2019) according to the manufacturer’s instructions. The blood in mice was collected, and the serum was separated after centrifugation at 3,000 rpm for 20 min within 2 h after self-coagulation. The microwell plate was coated with mouse vascular endothelial growth factor VEGF to make a solid phase antibody. The sample was added to the coated microwell and combined with the HRP-labeled detection antibody. After washing, the substrate TMB was added to coloring. TMB is converted into blue under the catalysis of HRP enzyme, and change to yellow under the action of nucleic acid. Measure the OD value with a microplate reader at 450 nm wavelength.

### Statistical Analysis

All values are expressed as mean ± standard deviation. A comparison of the results was performed with one-way ANOVA and Tukey’s multiple comparison tests (GraphPad Prism software 5.0, La Jolla, CA, United States). Statistically significant differences between groups were defined as *p*-values less than 0.05.

## Results

### Carrimycin and Monomeric Isovalerylspiramycin I Inhibit the Viability of Hepatocellular Carcinoma Cells

**FIGURE 1:**
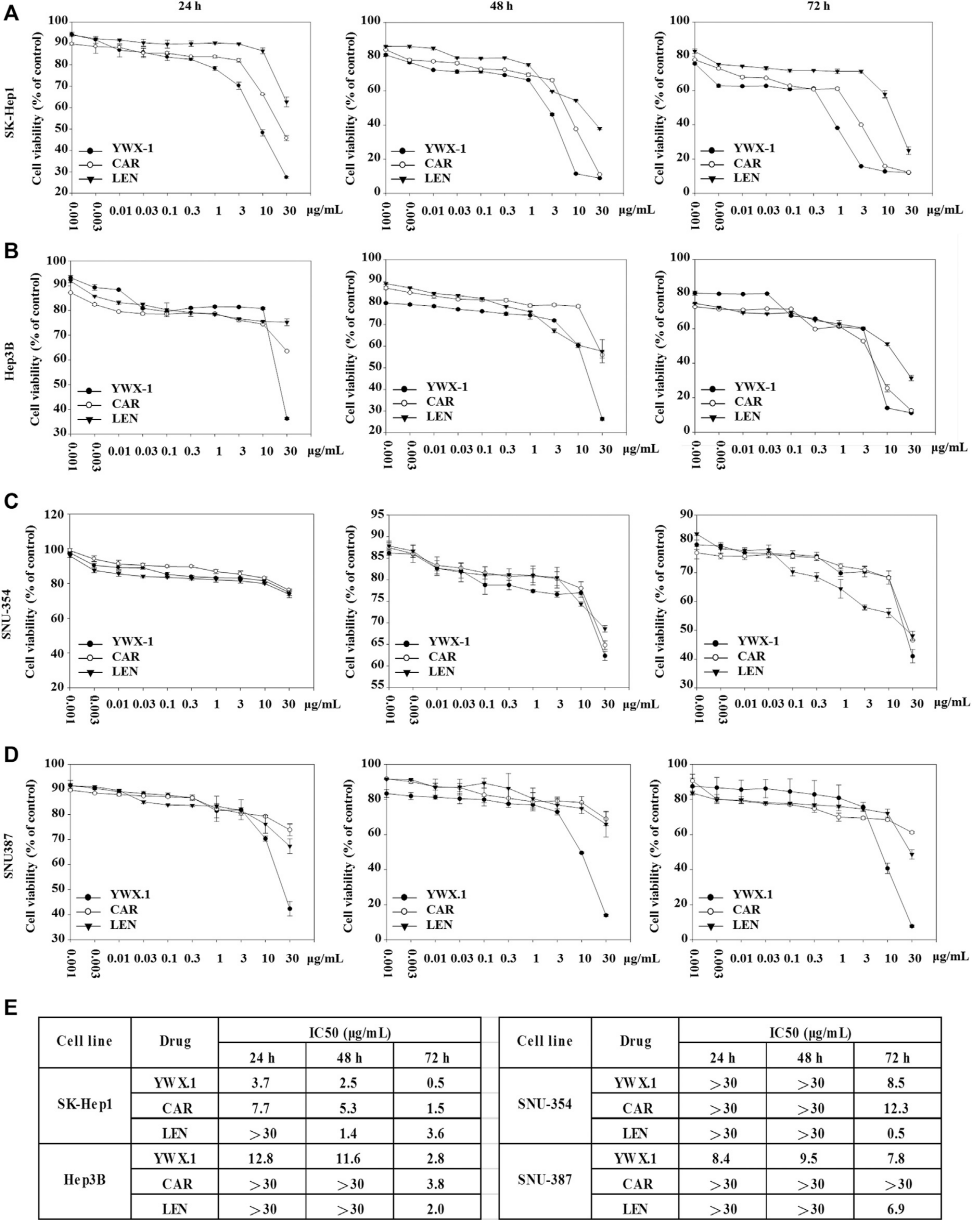
Carrimycin (CAR) and monomeric isovalerylspiramycin I (YWX-1) inhibit the viability of hepatocellular carcinoma cells. The hepatocellular carcinoma cells **(A)** SK-Hep1, **(B)** Hep3B, **(C)** SNU-354, and **(D)** SNU-387 were incubated with various concentrations of carrimycin and monomeric isovalerylspiramycin I at 24, 48, 72 h, and their viability were then determined by MTT assay. **(E)** IC50 value of carrimycin, monomeric isovalerylspiramycin I, and lenvatinib in SK-Hep1, Hep3B, SNU-354, and SNU-387 cells.

To detect the effect of carrimycin, monomeric isovalerylspiramycin I, and lenvatinib on hepatocellular carcinoma cells, we used the SK-Hep1, Hep3B, SNU-354, and SNU-387 cell lines to perform cell viability assay. Cells were treated with increasing concentrations of drug for 24, 48, and 72 h, and the cell viability was determined by MTT assay. At 24 h, compared with 0.001 μg/ml drug treatment groups, carrimycin, monomeric isovalerylspiramycin I, and lenvatinib have a significant inhibitory effect on cell viability. At 48 h and 72 h, the inhibitory effect of the three drugs was more obvious in SK-Hep1, Hep3B, SNU-354, and SNU-387 cell lines (Figures 1A–D). *In vitro* activities of the carrimycin, monomeric isovalerylspiramycin I, and lenvatinib were summarized in Supplementary Figure S1E.

### Carrimycin and Monomeric Isovalerylspiramycin I Inhibit Colony Formation of Hepatocellular Carcinoma Cells

**FIGURE 2:**
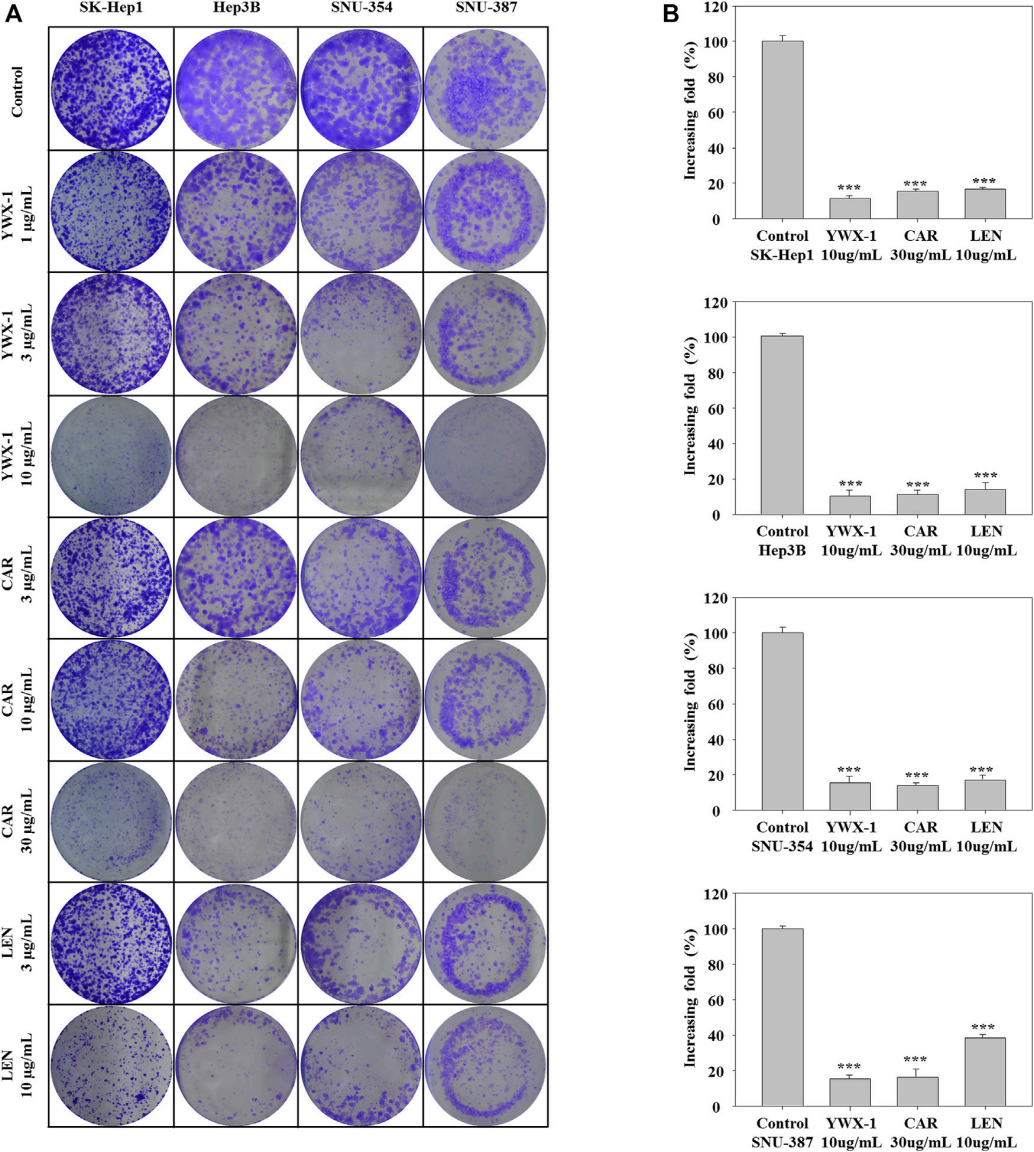
Carrimycin (CAR) and monomeric isovalerylspiramycin I (YWX-1) inhibit the colony formation of hepatocellular carcinoma cells. **(A)** SK-Hep1, Hep3B, SNU-354, and SNU-387 cells were treated with different concentrations of carrimycin and monomeric isovalerylspiramycin I. Cell proliferation was measured by colony formation assay. **(B)** Statistically significant differences are indicated when compared with control group: *p < 0.05, **p < 0.01, ***p < 0.001, Data represented as mean ± standard deviation of three independent experiments (n = 3).

We used colony formation experiments to further study cell proliferation. Compared with the control group, 30 μg/ml carrimycin, 10 μg/ml monomeric isovalerylspiramycin I, and 10 μg/ml lenvatinib can significantly inhibit cell colony formation, and the inhibitory effects of the three drugs are similar (Figures 2A,B). In SK-Hep1, Hep3B, and SNU-354 cells, the inhibition rates of monomeric isovalerylspiramycin I, carrimycin, and lenvatinib were greater than 80%. In SNU-387 cells, the inhibition rates of monomeric isovalerylspiramycin I and carrimycin were greater than 80%, and the inhibition rate of lenvatinib was approximately 60%.

### Carrimycin and Monomeric Isovalerylspiramycin I Inhibit DNA Replication in Hepatocellular Carcinoma Cells

**FIGURE 3:**
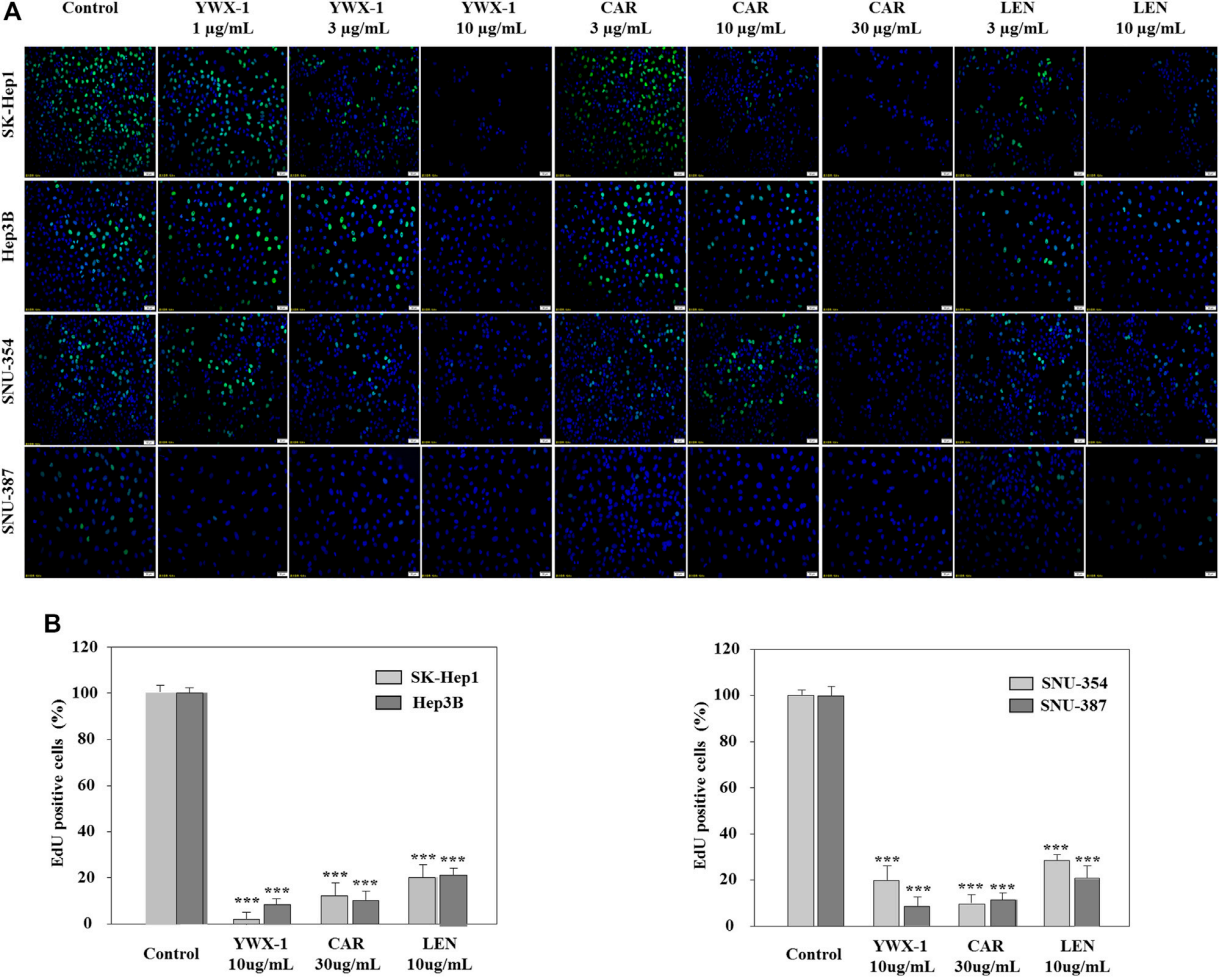
Carrimycin (CAR) and monomeric isovalerylspiramycin I (YWX-1) inhibit DNA replication in hepatocellular carcinoma cells. **(A)** SK-Hep1, Hep3B, SNU-354, and SNU-387 cells were treated with different concentrations of carrimycin and monomeric isovalerylspiramycin I. Cell proliferation was measured by EdU assays. The EdU-labeled replicating cells were examined under a fluorescence microscope (scale bar, 50 μm). **(B)** Statistically significant differences are indicated when compared with control group. Data represented as mean ± standard deviation of three independent experiments (n = 3).

To determine whether carrimycin, monomeric isovalerylspiramycin I, and lenvatinib could affect DNA replication, the EdU incorporation assay was performed. For all 4 cell lines, incorporation of EdU in DNA was significantly reduced after treatment with 30 μg/ml carrimycin, 10 μg/ml monomeric isovalerylspiramycin I, or 10 μg/ml lenvatinib compared with the negative control groups (Figure 3A). The inhibitory effect of carrimycin and monomeric isovalerylspiramycin I was similar to that of lenvatinib. As shown in Figure 3B, in the SK-Hep1, Hep3B, SNU-354 and SNU-387 lines, compared with the control group, the monomer isovalerylspiramycin I, carrimycin and levatinib all had inhibitory effects. The inhibitory activity of carrimycin, monomeric isovalerylspiramycin I and lenvatinib were similar.

### Carrimycin and Monomeric Isovalerylspiramycin I Suppress the Migration of Hepatocellular Carcinoma Cells

**FIGURE 4:**
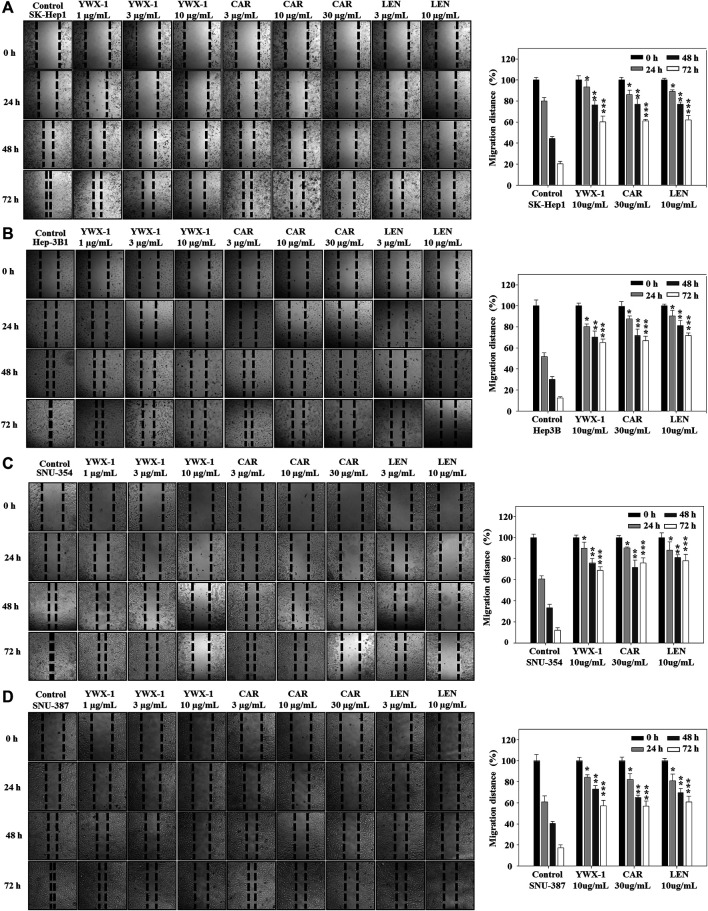
Carrimycin (CAR) and monomeric isovalerylspiramycin I (YWX-1) suppress the migration of hepatocellular carcinoma cells. **(A–D)** SK-Hep1, Hep3B, SNU-354, and SNU-387 cells were treated with different concentrations of carrimycin and monomeric isovalerylspiramycin I. Migration was observed by an optical microscope (×200 magnification). Statistically significant differences are indicated when compared with control group. Data represented as mean ± standard deviation of three independent experiments (n = 3).

Inhibiting the migration of hepatocellular carcinoma cells can effectively inhibit the spread of tumors. Therefore, we evaluated the migration potential of the hepatocellular carcinoma lines in the presence of the three agents. Figures 4A–D show that migration was significantly suppressed in the 4 cell lines after treatment with 30 μg/ml carrimycin, 10 μg/ml monomeric isovalerylspiramycin I, and 10 μg/ml lenvatinib. The histograms show that the inhibitory effects of the agents on migration were similar.

### Carrimycin and Monomeric Isovalerylspiramycin I Inhibit the Invasion of Hepatocellular Carcinoma Cells

**FIGURE 5:**
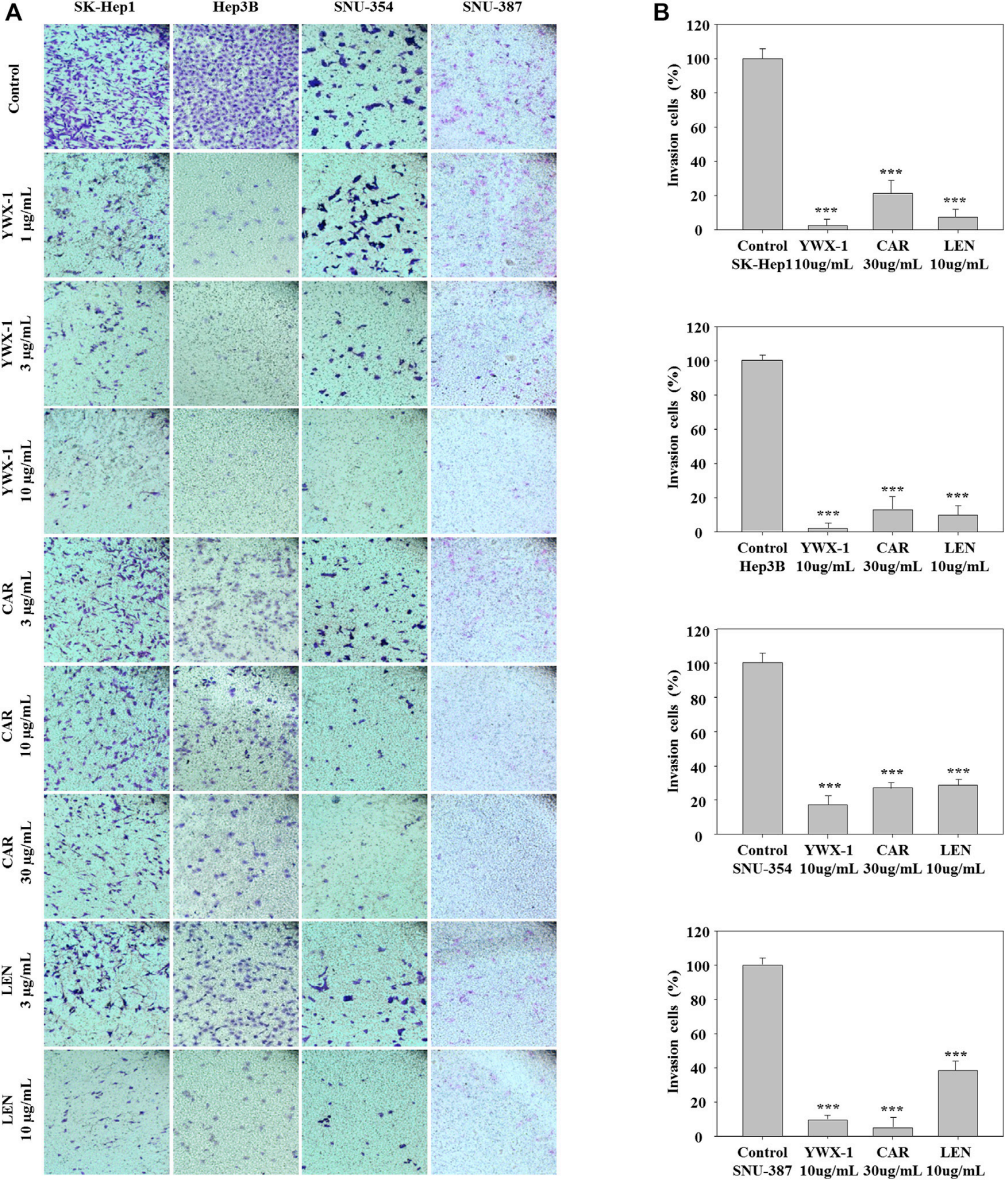
Carrimycin (CAR) and monomeric isovalerylspiramycin I (YWX-1) inhibit the invasion of hepatocellular carcinoma cells. **(A)** SK-Hep1, Hep3B, SNU-354, and SNU-387 cells were treated with different concentrations of carrimycin and monomeric isovalerylspiramycin I. Cell invasion was observed by an optical microscope (×200magnification). **(B)** Statistically significant differences are indicated when compared with control group. Data represented as mean ± standard deviation of three independent experiments (n = 3).

Inhibiting the invasion of hepatocellular carcinoma cells can effectively inhibit the spread of tumors, too. Therefore, we also evaluated the invasion potential of the hepatocellular carcinoma lines in the presence of the three agents. Figure 5A indicates that invasion potential was significantly inhibited in the 4 lines when treated with 30 μg/ml carrimycin, 10 μg/ml monomeric isovalerylspiramycin I, and 10 μg/ml lenvatinib compared with the negative control groups. The histograms show that the inhibitory effects of monomeric isovalerylspiramycin I, carrimycin, and lenvatinib on invasive potential were similar in all 4 cell lines (Figure 5B).

### Carrimycin and Monomeric Isovalerylspiramycin I Promote Apoptosis of Hepatocellular Carcinoma Cells

**FIGURE 6:**
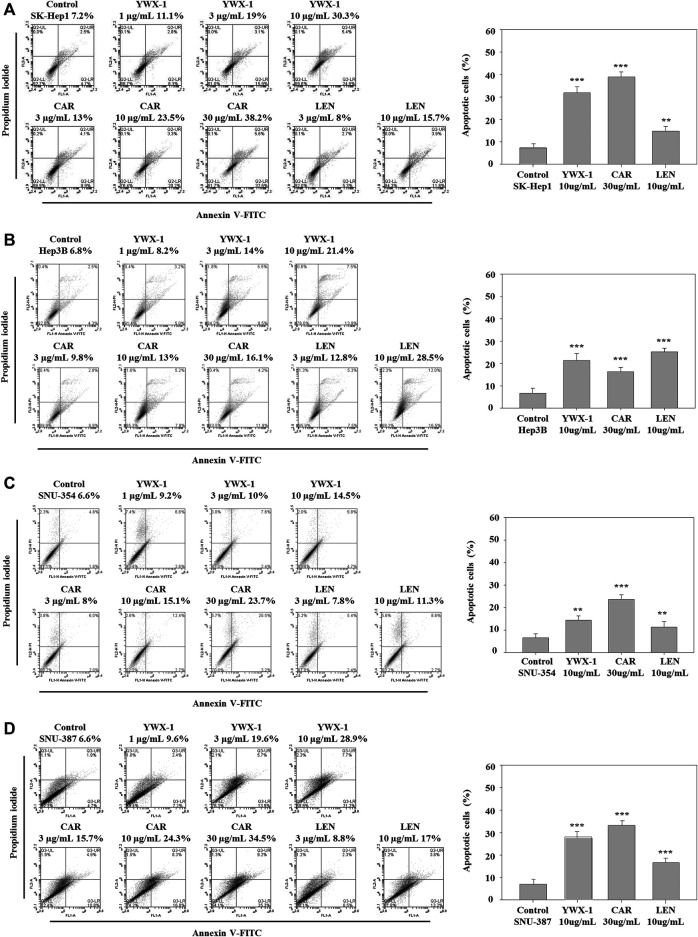
Carrimycin (CAR) and monomeric isovalerylspiramycin I (YWX-1) promote apoptosis of hepatocellular carcinoma cells. **(A–D)** SK-Hep1, Hep3B, SNU-354, and SNU-387 cells were treated with different concentrations of carrimycin and monomeric isovalerylspiramycin I, and subsequently, the apoptotic cells were detected by Annexin V-FITC/PI double staining assay. The percentages of four hepatocellular carcinoma apoptotic cells were calculated. Statistically significant differences are indicated when compared with control group. Data represented as mean ± standard deviation of three independent experiments (n = 3).

Promoting apoptosis of tumor cells can effectively alleviate the development and reduce the size of tumors. Figure 6 shows that the rate of apoptosis increased in each cell line after treatment with carrimycin, monomeric isovalerylspiramycin I, and lenvatinib compared with the negative control group. Figure 6 shows the apoptosis rates were 38.2, 30.3 and 15.7% in SK-Hep1 cells (Figure 6A), 16.1, 21.4, and 28.5% in Hep3B cells (Figure 6B), 23.7, 14.5 and 11.3% in SNU-354 cells (Figure 6C), and 34.5, 28.9, 17% in SNU-387 cells (Figure 6D) after treatment with 30 μg/ml carrimycin, 10 μg/ml monomeric isovalerylspiramycin I and 10 μg/ml lenvatinib, respectively. As detected by flow cytometry, the apoptosis rate of SK-Hep1, SNU-354, Hep3B and SNU-387 cells was promoted with the treament with carrimycin, monomeric isovalerylspiramycin I and lenvatinib compared with the control groups. The pro-apoptotic rates of carrimycin, monomeric isovalerylspiramycin I and lenvatinib were similar. The histograms show that carrimycin and monomeric isovalerylspiramycin I could promote apoptosis of hepatocellular carcinoma cells.

### Carrimycin and Monomeric Isovalerylspiramycin I Inhibit the Expression of VEGF and PD-L1 Protein in Hepatocellular Carcinoma Cells

**FIGURE 7:**
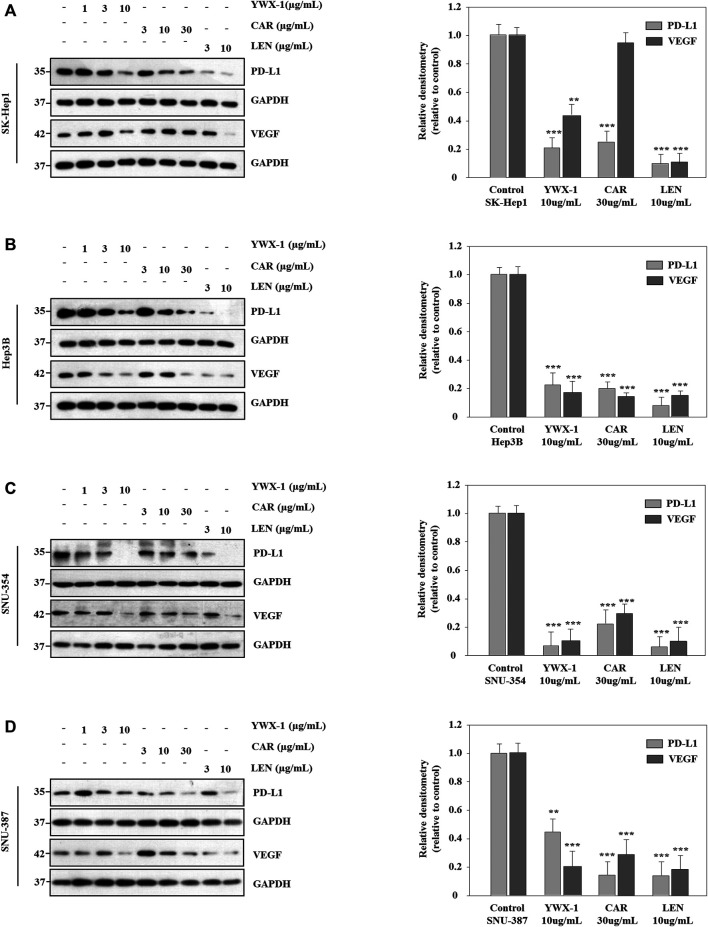
Carrimycin (CAR) and monomeric isovalerylspiramycin I (YWX-1) inhibit the expressions of VEGF and PD-L1 protein in hepatocellular carcinoma cells. **(A–D)** SK-Hep1, Hep3B, SNU-354, and SNU-387 cells were treated with different concentrations of carrimycin and monomeric isovalerylspiramycin I. The protein levels of VEGF and PD-L1 were analyzed by western blotting. GAPDH level was used as a loading control. Statistically significant differences are indicated when compared with control group. Data represented as mean ± standard deviation of three independent experiments (n = 3).

VEGF is an important marker of angiogenesis, and PD-L1 is an immune cell checkpoint-related protein. Therefore, we analyzed the expression of these two proteins by western blot. Figure 7 indicates that the expressions of PD-L1 protein levels were significantly lower after treatment with the three agents in all 4 lines compared with the negative control groups. And the expressions of VEGF protein levels were significantly lower after treatment with the monomer isovalerylspiramycin I and levatinib in all 4 cell lines compared with the negative control groups. However, carrimycin has no significant effect on VEGF in Sk-Hep1 cells (Figures 7A–D).

### Carrimycin and Monomeric Isovalerylspiramycin I Inhibit the Levels of VEGF and PD-L1 mRNA in Hepatocellular Carcinoma Cells

**FIGURE 8:**
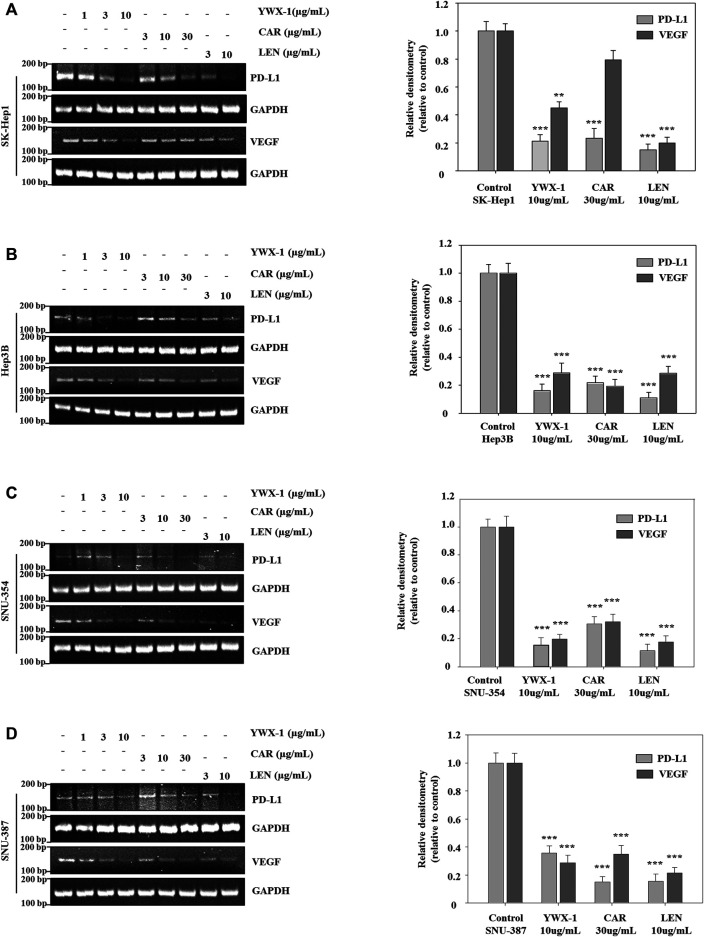
Carrimycin (CAR) and monomeric isovalerylspiramycin I (YWX-1) inhibit the levels of VEGF and PD-L1 mRNA in hepatocellular carcinoma cells. **(A–D)** SK-Hep1, Hep3B, SNU-354, and SNU-387 cells were treated with different concentrations of carrimycin and monomeric isovalerylspiramycin I. The mRNA levels of *VEGF* and *PD-L1* were analyzed by RT-PCR. *GAPDH* level was used as a loading control. Statistically significant differences are indicated when compared with control group: *p < 0.05, **p < 0.01, ***p < 0.001, Data represented as mean ± standard deviation of three independent experiments (n = 3).

Next, we test the effect of carrimycin, monomeric isovalerylspiramycin I, and lenvatinib on *VEGF* and *PD-L1* mRNA levels in SK-Hep1, SNU-354, Hep3B and SNU-387 cells. As showed in Figures 8A–D, compared with the control group, carrimycin, monomeric isovalerylspiramycin I, and lenvatinib can inhibit *VEGF* and *PD-L1* mRNA levels in a dose-dependent manner, and the inhibitory effects of 30 μg/ml carrimycin, 10 μg/ml monomeric isovalerylspiramycin I, and 10 μg/ml lenvatinib on *VEGF* and *PD-L1* mRNA levels were similar. The primer sequence in Supplementary Table S1.

### Carrimycin and Monomeric Isovalerylspiramycin I Inhibit the Growth of SK-Hep1 Cells in a Subcutaneous Xenograft Tumor Model

**FIGURE 9:**
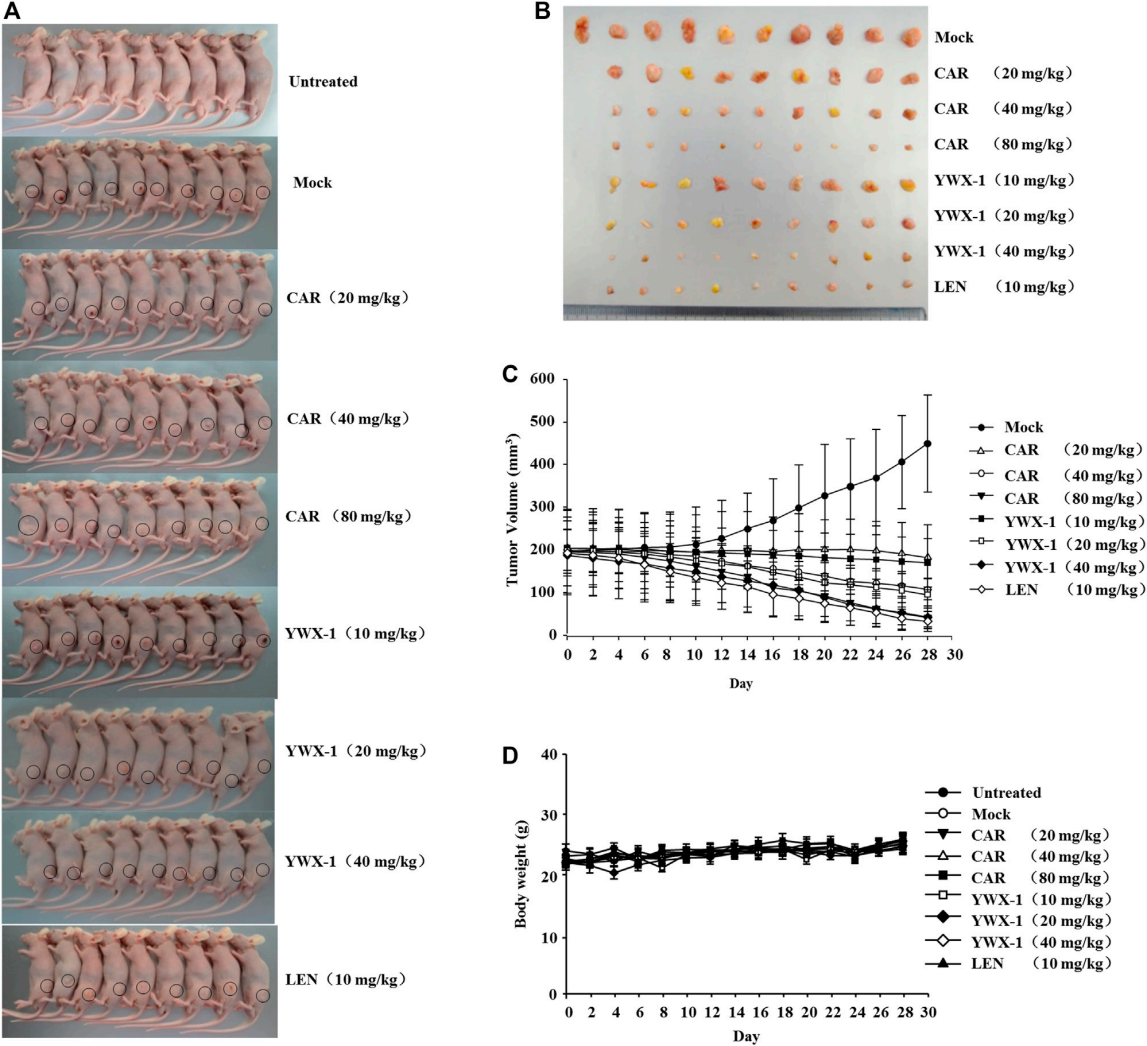
Carrimycin (CAR) and monomeric isovalerylspiramycin I (YWX-1) inhibit the growth of SK-Hep1 cells in a subcutaneous xenograft tumor model. **(A)** Representative images of xenografts. **(B)** The representative tumor was harvested 24 h after the last treatment and the isolated tumors were photographed. **(C)** SK-Hep1 cells were implanted subcutaneously in the flanks of nude mice. Tumor volume was calculated every 2 days using the equation: (width)^2^ × length/2. **(D)** The bodyweight of each group of mice was measured using a digital balance.

**FIGURE 10:**
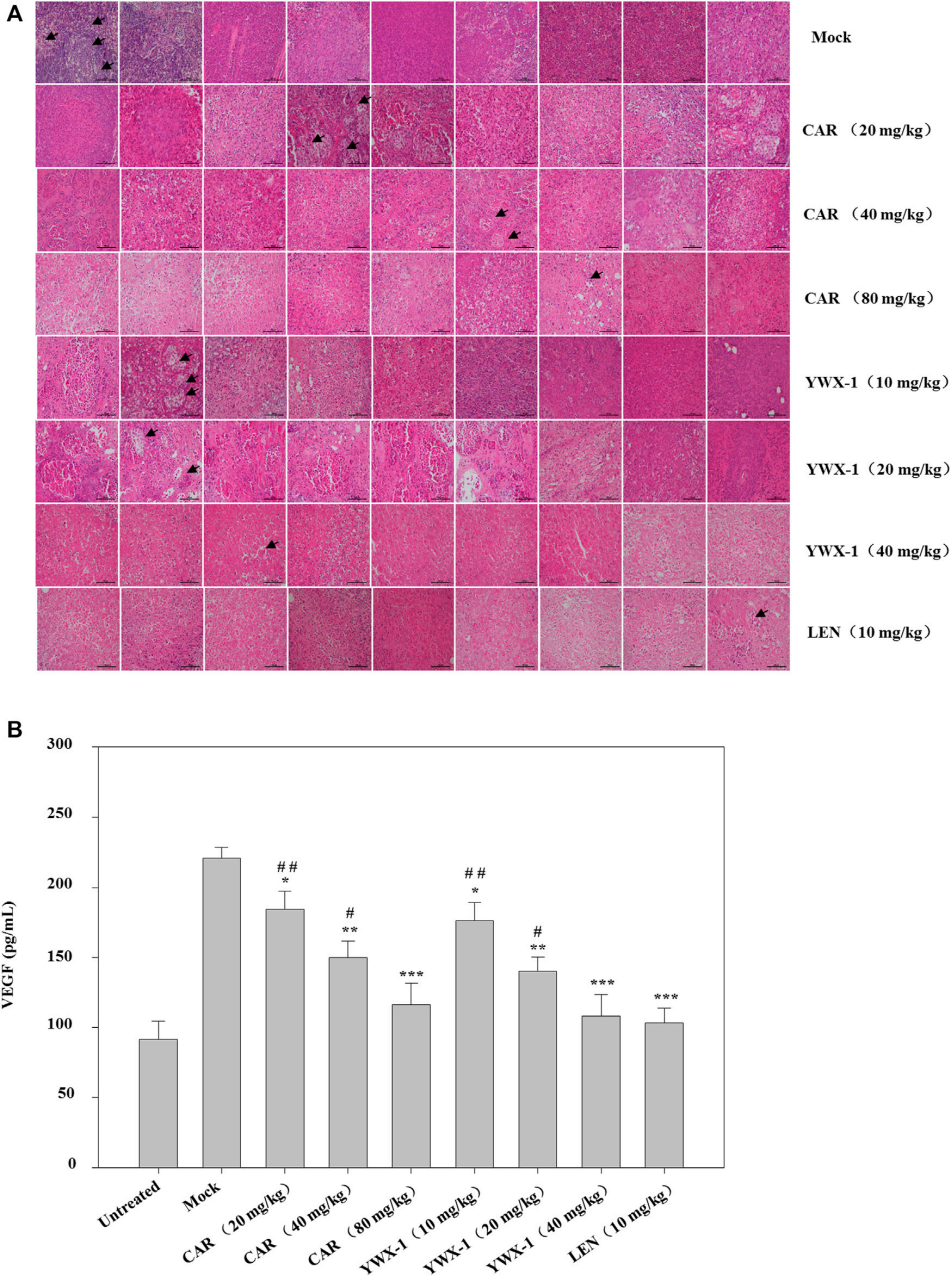
Effect of carrimycin (CAR) and monomeric isovalerylspiramycin I (YWX-1) on the histopathology and VEGF secretion in a subcutaneous xenograft tumor model. **(A)** Tumor sections were stained with H&E. Histological alterations are indicated by arrows. (scale bar, 100 μm). **(B)** The concentration of VEGF in mice serum was detected by ELISA analysis. Data were presented as the mean ± standard deviation of three independent experiments (n=3). * *p* < 0.05, ** *p* < 0.01, *** *p* < 0.001, compared with the mock group; # *p* < 0.05, ## *p* < 0.01, ### *p* <0.001 compared with the positive control drug lenvatinib (LEN) 10 mg/kg group.

Given that carrimycin and monomeric isovalerylspiramycin I inhibited the proliferation of tumor cells *in vitro*, we investigated whether these results could be translated *in vivo*. Thus, we developed an HCC xenograft model using SK-Hep1 cells in nude mice. The tumor volumes of the treated groups were significantly reduced compared with the mock group (Figures 9A,B). Compared with the positive control drug lenvatinib (10 mg/kg dose), there were similarities results with crimycin at the 80 mg/kg dose and the monomeric isovalerylspiramycin I at the 40 mg/kg dose. As shown in Figure 9C, after 28 days, carrimycin dose of 20, 40, and 80 mg/kg resulted in tumor inhibition rates of 59.31, 75.88, 90.62%, respectively, and monomeric isovalerylspiramycin I dose of 10, 20, and 40 mg/kg resulted in 62.15, 78.74, 90.47% tumor inhibition, respectively. The positive control group treated with 10 mg/kg lenvatinib showed a tumor inhibition rate of 92.61%. Figure 9D indicates that the body weights of the mice did not change. The tumor tissues were embedded in paraffin and stained with hematoxylin and eosin for histological examination and compared with the mock control group. After staining the tumor tissue with HE (Figure 10A), the tumor tissues in the model group were poor differentiation, large atypia, invasive or exogenous growth, and the boundary between them and surrounding tissue was not clear, as shown by the arrows. After drug treatment, the tumor tissues were well differentiation, with small atypia, rare mitotic, without pathological mitotic figures, expansion or exogenous growth, the boundary with the surrounding tissue is clear. The tumor tissues in the 20 mg/kg group of carrimycin and the 10 mg/kg group of monomeric isovalerylspiramycin I showed peripheral invasive growth. However, compared with the mock mice, the infiltrating growth around the tumor tissues was weakened by carrimycin (40 mg/kg and 80 mg/kg doses), monomeric isovalerylspiramycin I (20 mg/kg and 40 mg/kg doses), and lenvatinib (10 mg/kg dose), the boundary with the periphery became clear and the tumor size was significantly reduced. Using the ELISA, it was observed from Figure 10B that carrimycin, monomeric isovalerylspiramycin I, and lenvatinib treatment significantly reduced the concentration of VEGF in mouse serum. Compared with the positive control drug lenvatinib, the effect of carrimycin and monomer isovalerylspiramycin I on the concentration of VEGF was similar.

### Carrimycin and Monomeric Isovalerylspiramycin I Inhibit the Growth of H22 Cells in an Orthotopic Liver Transplantation Model

**FIGURE 11:**
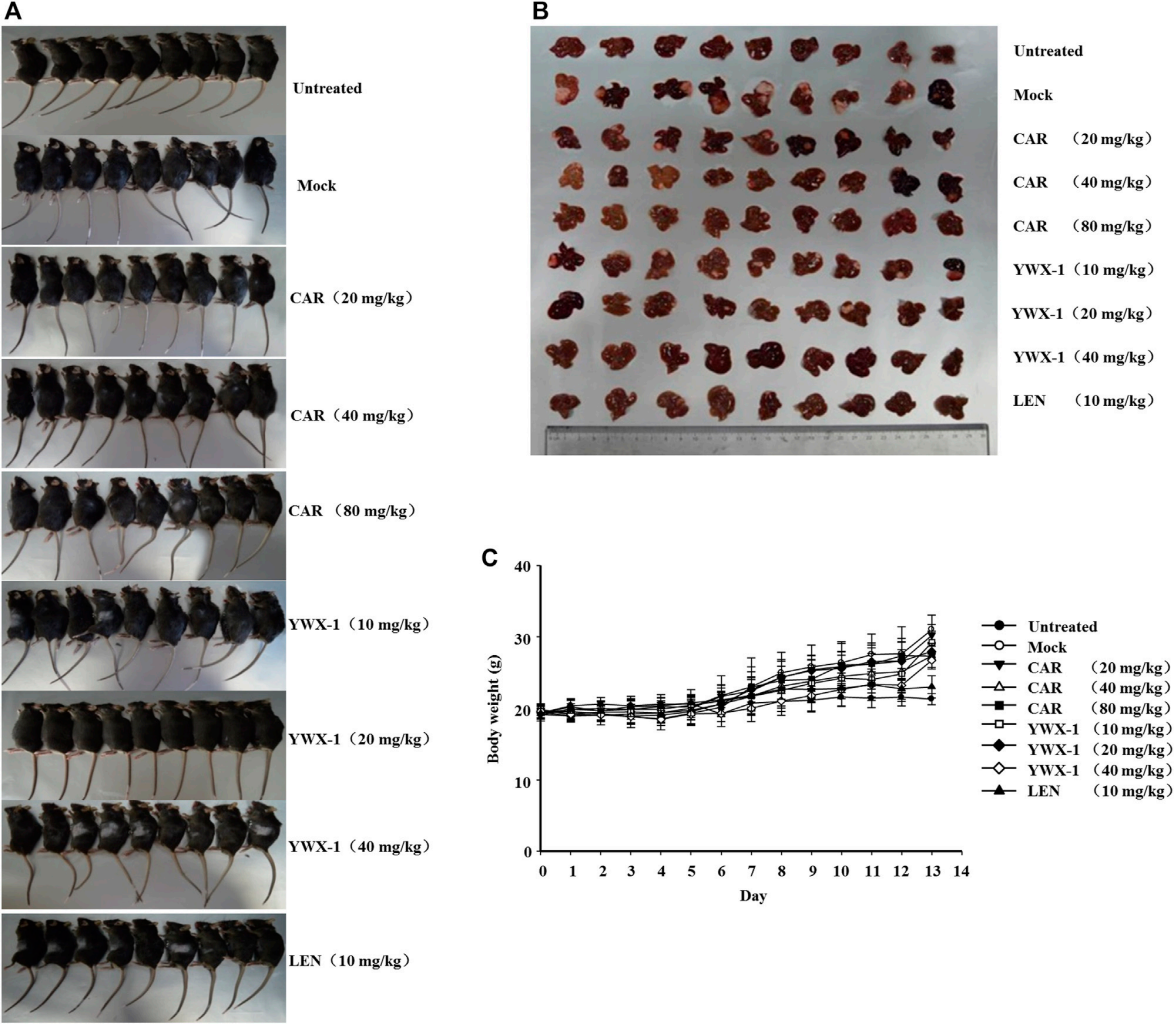
Carrimycin (CAR) and monomeric isovalerylspiramycin I (YWX-1) inhibit the growth of H22 cells in an orthotopic liver transplantation model. **(A)** Representative images of orthotopic liver transplantation model. **(B)** The representative tumor was harvested 24 h after the last treatment and the isolated tumors were photographed. **(C)** The ascites H22 cells were injected into the mouse liver. Bodyweight changes of each group of mice.

**FIGURE 12:**
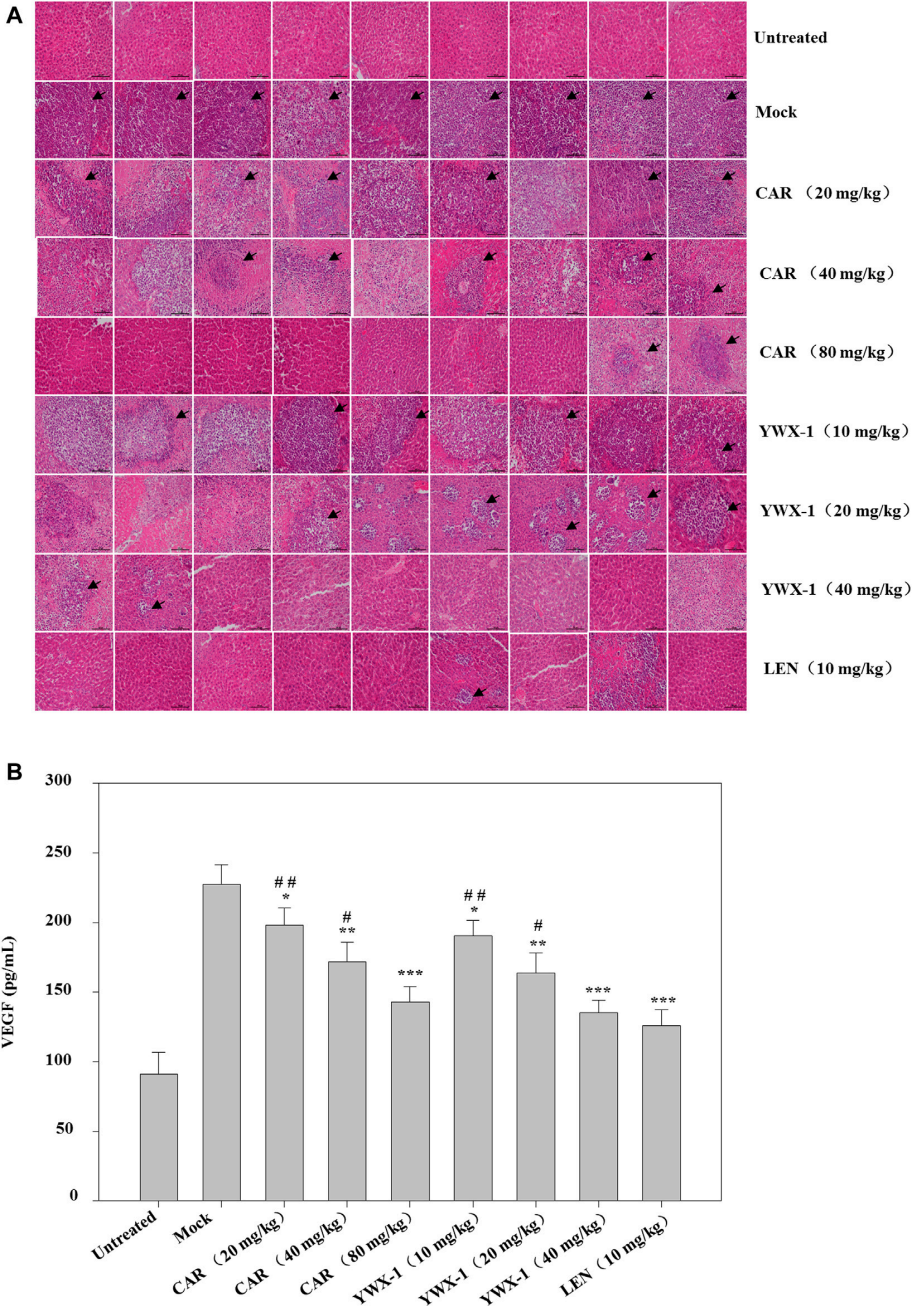
Effect of carrimycin (CAR) and monomeric isovalerylspiramycin I (YWX-1) on the histopathology and VEGF secretion in an orthotopic liver transplantation model. **(A)** Tumor sections were stained with H&E. Histological alterations are indicated by arrows. (scale bar, 100 μm). **(B)** The concentration of VEGF in mice serum was detected by ELISA analysis. Data were presented as the mean ± standard deviation of three independent experiments (n=3). *p < 0.05, **p < 0.01, ***p < 0.001, compared with the mock group; ^#^ *p* < 0.05, ^##^ *p* < 0.01, ^###^ *p* < 0.001 compared with the positive control drug lenvatinib (LEN) 10 mg/kg group.

We examined the *in vivo* efficacy of carrimycin and monomeric isovalerylspiramycin I in an orthotopic liver transplantation model for HCC. Figures 11A,B show the representative mice and the transplanted tumor pictures of orthotopic liver transplantation model. Compared with the positive control drug lenvatinib (10 mg/kg dose), there were similarities results with crimycin at the 80 mg/kg dose and the monomeric isovalerylspiramycin I at the 40 mg/kg dose. There were no differences in body weight between the groups of mice (Figure 11C). After staining the tumor tissue with HE (Figure 12A), it was observed that the tumors in the mock group increased in mass and developed large. The transplanted tumor was completely or partially enveloped with the surrounding liver tissue, and there were nodules with different sizes on the surface, mainly with invasive growth, as shown by the arrows. The tumor tissues in the 20 mg/kg group of carrimycin and the 10 mg/kg group of monomeric isovalerylspiramycin I showed peripheral invasive growth. However, compared with the mock mice, the infiltrating growth around the tumor tissues was weakened by carrimycin (40 mg/kg and 80 mg/kg doses), monomeric isovalerylspiramycin I (20 mg/kg and 40 mg/kg doses), and lenvatinib (10 mg/kg dose), the boundary with the periphery became clear and the tumor size was significantly reduced. Furthermore, Figure 12B shows that carrimycin, monomeric isovalerylspiramycin I, and lenvatinib significantly reduced the concentration of VEGF in mice serum compared with the mock mice.

## Discussion

Anti-tumor antibiotic drugs consist of anthracyclines, macrolides, glycopeptides, phenylalanine pyrrole, enediyne and quinoxaline, with high bioavailability and biosafety. Some macrolides antibiotics were reported to have anti-tumor effects. Immunosuppressor rapamycin inhibits tumor progression by targeting mammalian target of rapamycin (mTOR) (Foster and Fingar, 2010). Epothilone analogs inhibit tumor progression by down-regulating the phosphorylation of AKT, ERK and p38 (Edelman and Shvartsbeyn, 2012; Li et al., 2016). Another macrolides antibiotic tanespimycin (17-AAG) inhibits tumor progression by suppressing p-ERK and p-AKT levels (Sato et al., 2000; Dou et al., 2005). It has been also reported that dynactin, a macrolide antibiotic, from *Streptomyces puniceus* AS13, is an effective cytostatic anti-cancer agent that inhibits the proliferation of human cancers without inducing cell death (Hussain et al., 2019). Since few studies have reported the possible mechanism of carrimycin inhibiting tumors, this research contributes to our understanding of target and molecular mechanism of carrimycin to inhibit tumor progression in hepatocellular carcinoma.

Hepatocellular carcinoma is characterized by its association with chronic inflammation and an immunosuppressive tumor microenvironment, which makes immunotherapy an approach for new drug development (Chang et al., 2017). Upregulation of PD-L1 is common in HCC and has been correlated with both poor prognosis and tumor progression. Among immune checkpoints, the PD-1/PD-L1 pathway has emerged as a strong target for HCC (Taube et al., 2014). Thus, understanding the mechanism by which cancer cells regulate PD-L1 expression is invaluable for developing therapeutic strategies and predicting the clinical outcome of cancer patients being treated with immunotherapy (Koh et al., 2016). Consistently, anti-PD-1 or anti-PD-L1 antibodies blocking the binding between PD-1 and PD-L1 have been reported to promote marked antitumor immunity, and have risen to the forefront of immunotherapy due to their notable clinical efficacy in melanoma and non-small cell lung cancer clinical trials (Xue et al., 2017). PD-L1 expression was significantly associated with an unfavorable clinical outcome in terms of PFS and OS of patients (Koh et al., 2016). Immunotherapies with checkpoint inhibitor PD-L1/PD-1 antibodies have shown encouraging results in patients with NSCLC (Hirsch et al., 2017). In our study, carrimycin and monomeric isovalerylspiramycin I could effectively inhibit PD-L1 expression in hepatocellular carcinoma cells.

Additionally, hepatocellular carcinoma is known to be a highly vascularized solid tumor in which angiogenesis plays an important role in tumor progression, high recurrence rates, and poor survival. Vascular endothelial growth factor (VEGF) is a major growth factor that promotes HCC progression by regulating endothelial cell proliferation, migration, differentiation, and vessel formation (Pang and Poon, 2006). VEGF expression can indirectly reflect the proliferation, migration, and angiogenesis of endothelial cells in a tumor and indicate tumor growth rate and metastasis tendency (Chehelcheraghi et al., 2019). Therefore, suppressing tumor angiogenesis by inhibiting VEGF is of great significance for the control of tumor growth and metastasis. In this study, carrimycin and monomeric isovalerylspiramycin I could effectively inhibit VEGF expression in hepatocellular carcinoma cells.

It has been reported that tumor occurrence and development are associated with abnormal cell proliferation, differentiation, and apoptosis. The following is a literature review of ten basic mechanisms of tumor occurrence and development: proliferation pathway activation, inactivation of genes that inhibit proliferation, immune evasion, activation of invasion and metastasis pathways, inflammation, unchecked DNA replication, induction of angiogenesis, genome instability, and mutation, resistance to apoptosis, and changes in energy metabolism (Hanahan and Weinberg, 2011; Sasahira and Kirita, 2018). Inhibiting the invasion and migration of liver cancer cells can inhibit the spread of tumors. Promoting the apoptosis of tumor cells can effectively alleviate the progression of tumors. Apoptosis is an autonomous and orderly death of cells controlled by specific genes that maintain the homeostasis of the internal environment. Tumor cells can acquire resistance to apoptosis in several ways, such as impaired signal transduction of death receptors, destruction of the balance between pro-apoptotic and anti-apoptotic proteins, decreased function of caspase, and impaired function of p53 (Pistritto et al., 2016). Our results showed that carrimycin and monomeric isovalerylspiramycin I could effectively inhibit proliferation, colony formation, cell migration, and invasion in a time-concentration-dependent manner, and induce apoptosis in hepatocellular carcinoma cells.

At present, a large number of studies have provided a basic basis for the clinical treatment of liver cancer, and liver cancer animal models have always been an important tool for clinical basic research work of liver cancer. The orthotopic transplantation model of liver cancer and the xenograft tumor model are used together to better simulate the occurrence, development, invasion and metastasis of human liver cancer *in vivo*. In the vitro pilot study, we chose the carrimycin concentration of 10, 20, 40, 80, 100 mg/kg, and monomeric isovalerylspiramycin I concentration of 10, 20, 40, 80, 100 mg/kg. We found that carrimycin of 80 mg/kg and monomeric isovalerylspiramycin I of 40 mg/kg had similar inhibitory effects on the concentration of VEGF, Therefore, in the formal experiment, we choose the concentration of lenvatinib to be 20, 40, 80 mg/kg, and the concentration of monomeric isovalerylspiramycin I to be 10, 20, 40 mg/kg. For lenvatinib, we referred to the papers and choose a dose of 10 mg/kg (Matsuki et al., 2018; Hoshi et al., 2019). Animal studies indicated that carrimycin and monomeric isovalerylspiramycin I could significantly inhibit tumor volume, tumor histopathology, and the concentration of VEGF in two mouse models for HCC. Long-term effects of carrimycin in tumor inhibition require more in-depth investigations. As mentioned above, the evidence supporting carrimycin and monomeric isovalerylspiramycin I as inhibitors of HCC is convincing. Future research will further explore the specific anti-tumor mechanism of carrimycin and monomeric isovalerylspiramycin I.

As for clinical translation, macrolide antibiotic rapamycin is limited in its application due to its poor water solubility, poor absorption capacity and low bioavailability (Liu et al., 2019). Preclinical studies revealed a tumor inhibition effect of its derivatives. However, the results of clinical trials were discouraging due to severe side effects and high toxicity. Clinical trials of macrolide antibiotics in combination with other drugs also showed poor therapeutic effect or severe side effects (Piha-Paul et al., 2015; Barata et al., 2019). In this study, carrimycin and its main monomer component isovalerylspiramycin I have significant anticancer effects similar to levatinib. It is reported that carrimycin has good bioavailability and high biosafety on phase I clinical trials, and carrimycin shows broad prospects in clinical applications.

## Conclusion

In conclusion, carrimycin and monomeric isovalerylspiramycin I inhibited the viability, colony formation, DNA replication, migration, invasion, and promoted apoptosis of HCC cells, and inhibited VEGF and PD-L1 protein expression, tumor growth, tumor histopathological alterations, and the concentration of VEGF in mouse serum. The mechanism may be related to the inhibition of the VEGF and/or PD-L1 signaling pathways. However, there are few in-depth studies on the mechanism of action of carrimycin and monomeric isovalerylspiramycin I. The mechanism of HCC inhibition by these agents will be explored in subsequent experiments. These data may provide a new treatment strategy for HCC that utilizes the inhibitory activity of carrimycin and monomeric isoprene I.

## Data Availability

The datasets presented in this study can be found in online repositories. The names of the repository/repositories and accession number(s) can be found below: https://www.jianguoyun.com/p/DV5Rz7YQ0tzeCRiauIwE.

## Abbreviations

CAR, carrimycin; HCC, hepatocellular carcinoma; LEN, lenvatinib; PD-1, programmed cell death-1; PD-L1, programmed cell death-ligand-1; VEGF, vascular endothelial growth factor; YWX-1, monomeric isovalerylspiramycin I.
