# Supplementary material for: Anti-Tumor Effects of Carrimycin and Monomeric Isovalerylspiramycin I on Hepatocellular Carcinoma in Vitro and in Vivo
Source: Front Pharmacol. 2021 Nov 26;12:774231. doi: 10.3389/fphar.2021.774231 (PMC8662527; doi:10.3389/fphar.2021.774231)
Supplement: Supplementary file 1 [file DataSheet1.PDF]

医学伦理审查报告

我校 Xuejun Jin 发表的论文题目为 Anti-tumor effects of carrimycin and monomeric isovalerylspiramycin I on hepatocellular carcinoma *in vitro* and *in vivo* 的研究，其研究中涉及的动物实验、临床患者的生物样品、私人疾病信息，我校伦理委员会对其进行了审查。

该课题前期研究内容严格遵循《赫尔辛基宣言》、世界卫生组织与国际医学科学组织理事会共同制定的《涉及人的生物医学研究国际伦理准则》、充分考虑动物福利以及国家对动物实验管理和伦理的要求，按照国家自然科学基金委员会有关规定进行科学研究。在本项目的实施过程中，能够严格做到知情同意、告知样品来源者生物样品的用途、处理方案与可能的补偿方式，保证样品来源的个人信息、医疗信息不被公开披露，在法律允许范围内尽一切努力保护样品来源者的个人医疗资料、疾病信息、生命信息和基因信息的隐私，能够遵守实验管理和伦理的各项要求。学校医学伦理委员会也将对项目的后续研究进行监督与审查，使其遵循上述原则与相关规定。

审查评议意见：

经我校医学伦理委员会审议，该研究的实验设计和实施方案充分考虑了安全性和公平性原则，研究内容不构成对受试者的伤害和风险，受试者的招募将完全基于自愿和知情同意原则，并尽最大限度保护受试者的隐私（或充分考虑动物福利以及非国家对动物实验管理和伦理的要求），研究内容和结果不存在利益冲突。

结论：

该研究中，受试者权利和利益得到了充分保护，对受试者不存在潜在风险（或者动物实验严格按照规程进行）。同意该项研究的现场工作按计划进行

延边大学医学院医学伦理委员会

2021 年 3 月 31 日
