# Supplementary figures and images for: Anti-Tumor Effects of Carrimycin and Monomeric Isovalerylspiramycin I on Hepatocellular Carcinoma in Vitro and in Vivo
Source: Front Pharmacol. 2021 Nov 26;12:774231. doi: 10.3389/fphar.2021.774231 (PMC8662527; doi:10.3389/fphar.2021.774231)

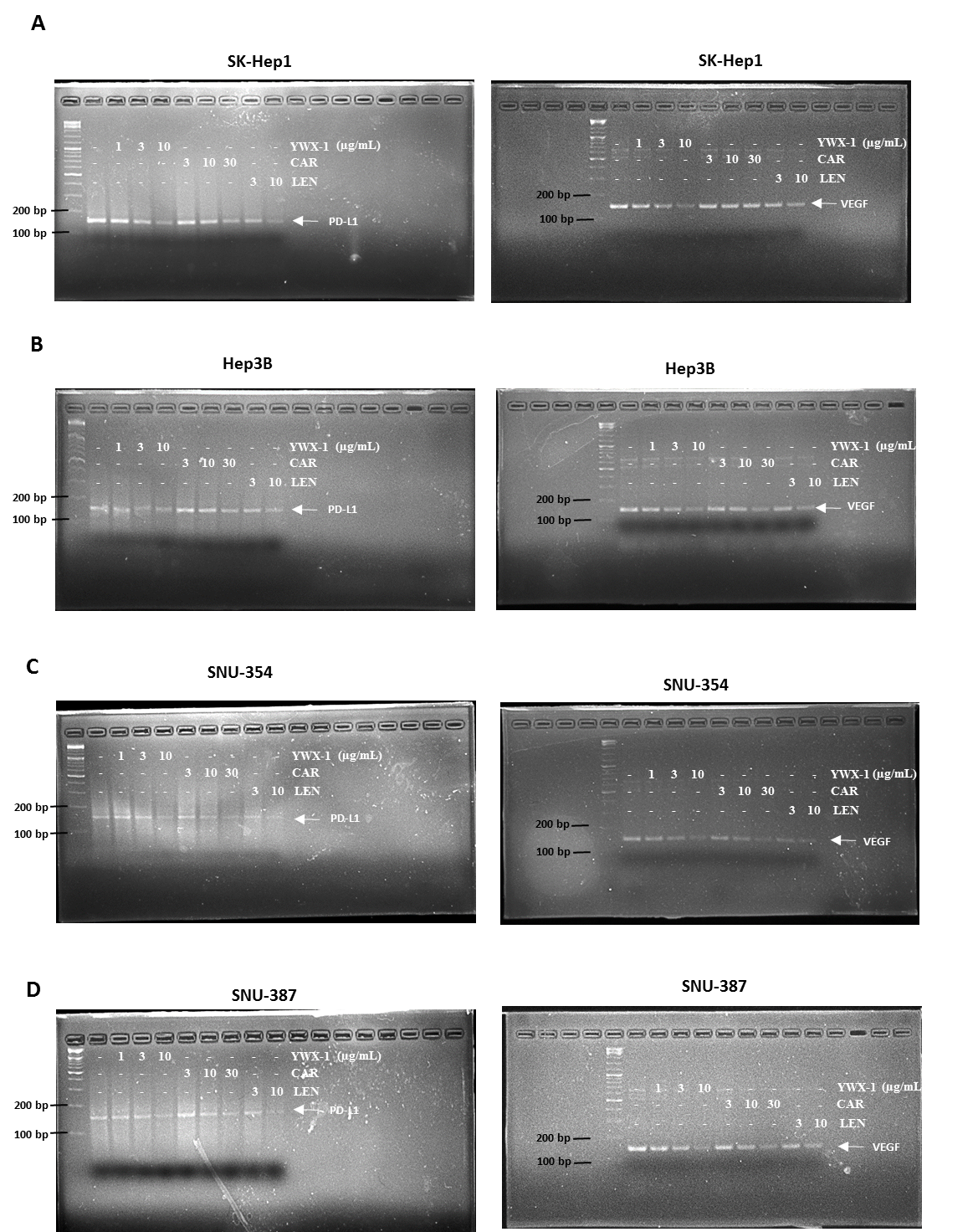


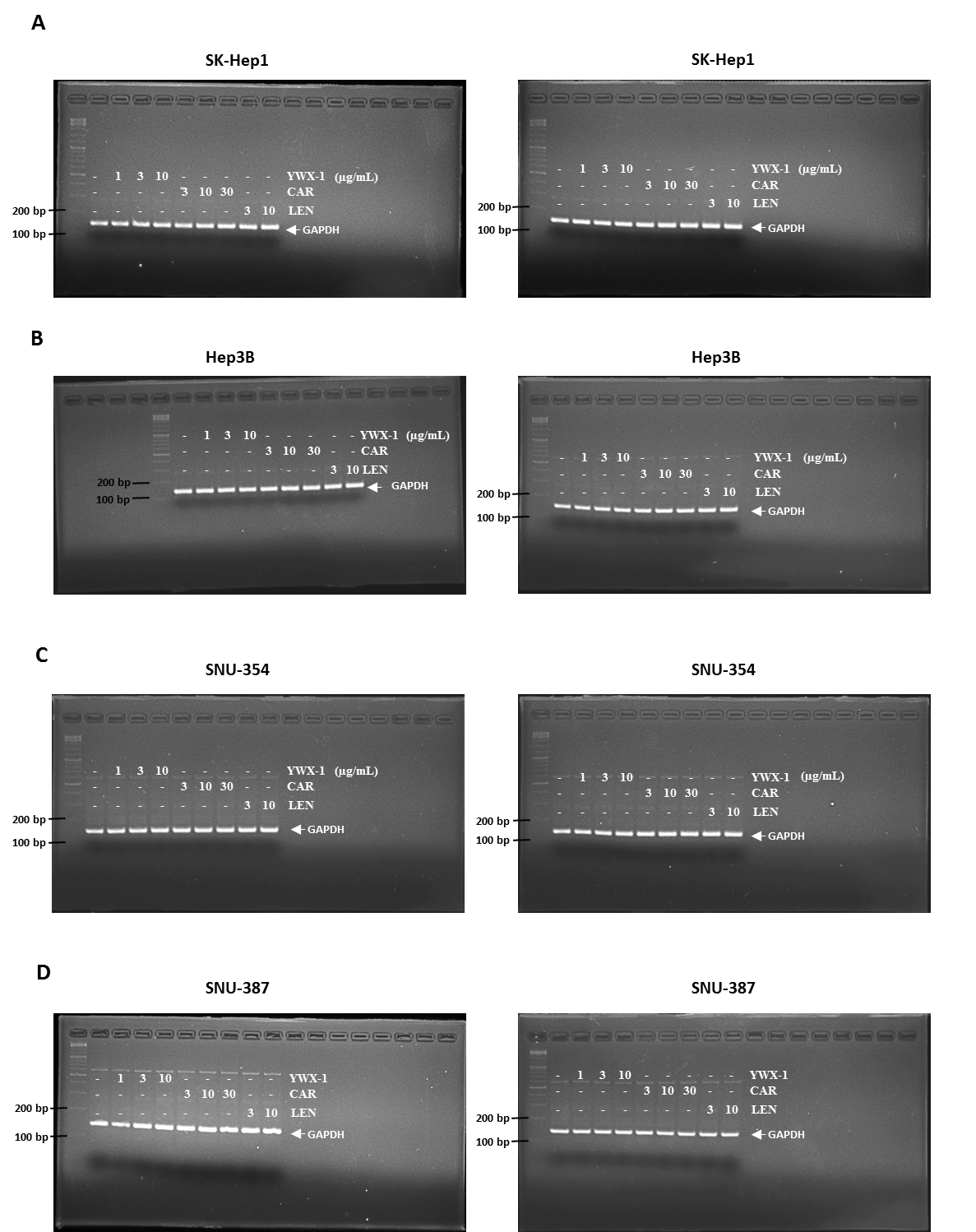

Supplement: Supplementary file 2 [file Table3.DOCX]
