# Supplementary material for: Anti-Tumor Effects of Carrimycin and Monomeric Isovalerylspiramycin I on Hepatocellular Carcinoma in Vitro and in Vivo
Source: Front Pharmacol. 2021 Nov 26;12:774231. doi: 10.3389/fphar.2021.774231 (PMC8662527; doi:10.3389/fphar.2021.774231)
Supplement: Supplementary file 3 [file Table4.DOCX]

**Table S1. Primers used in this study for RT-PCR**
